# Increased neutrophil extracellular trap formation in oligoarticular, polyarticular juvenile idiopathic arthritis and enthesitis-related arthritis: biomarkers for diagnosis and disease activity

**DOI:** 10.3389/fimmu.2024.1436193

**Published:** 2024-08-09

**Authors:** Hongxia Tang, Yucheng Zhong, Yali Wu, Yanmei Huang, Yi Liu, Jing Chen, Ting Xi, Yini Wen, Ting He, Shanshan Yang, Fan Liu, Runji Xiong, Runming Jin

**Affiliations:** ^1^ Department of Pediatrics, Union Hospital, Tongji Medical College, Huazhong University of Science and Technology, Wuhan, China; ^2^ Department of Rheumatology and Immunology, Wuhan Children’s Hospital, Tongji Medical College, Huazhong University of Science & Technology, Wuhan, China; ^3^ Department of Cardiovascular Surgery, Union Hospital, Tongji Medical College, Huazhong University of Science and Technology, Wuhan, Hubei, China; ^4^ Department of Pathogen Biology, School of Basic Medicine, Tongji Medical College, Huazhong University of Science and Technology, Wuhan, Hubei, China

**Keywords:** juvenile idiopathic arthritis, neutrophils, neutrophil extracellular traps, biomarkers, tumor necrosis factor-alpha

## Abstract

**Objective:**

Neutrophil extracellular traps (NETs) are important factors in initiating and perpetuating inflammation. However, the role of NETs in different subtypes of juvenile idiopathic arthritis (JIA) has been rarely studied. Therefore, we aimed to explore the ability of JIA-derived neutrophils to release NETs and the effect of TNF-α (tumor necrosis factor-alpha) inhibitors on NET formation both *in vitro* and *in vivo*, and evaluate the associations of NET-derived products with clinical and immune-related parameters.

**Methods:**

The ability of neutrophils to release NETs and the effect of adalimumab on NET formation was assessed via *in vitro* stimulation and inhibition studies. Plasma NET-derived products were detected to assess the incidence of NET formation *in vivo*. Furthermore, flow cytometry and western blotting were used to detect NET-associated signaling components in neutrophils.

**Results:**

Compared to those derived from HCs, neutrophils derived from patients with oligoarticular-JIA, polyarticular-JIA and enthesitis-related arthritis were more prone to generate NETs spontaneously and in response to TNF-α or PMA *in vitro*. Excessive NET formation existed in peripheral circulation of JIA patients, and elevated plasma levels of NET-derived products (cell-free DNA and MPO-DNA complexes) could accurately distinguish JIA patients from HCs and were positively correlated with disease activity. Multiple linear regression analysis showed that erythrocyte sedimentation rate and TNF-α levels were independent variables and were positively correlated with cell-free DNA concentration. Notably, TNF-α inhibitors could effectively prevent NET formation both *in vitro* and *in vivo*. Moreover, the phosphorylation levels of NET-associated kinases in JIA-derived neutrophils were markedly increased.

**Conclusion:**

Our data suggest that NETs might play pathogenic roles and may be involved in TNF-α-mediated inflammation in JIA. Circulating NET-derived products possess potential diagnostic and disease monitoring value. Furthermore, the preliminary results related to the molecular mechanisms of NET formation in JIA patients provide a theoretical basis for NET-targeted therapy.

## Introduction

Juvenile idiopathic arthritis (JIA) refers to a heterogeneous group of chronic childhood arthritis of unknown etiology that persist for more than 6 weeks and occur before the age of 16 (1). The International League of Associations for Rheumatology (ILAR) identified seven subtypes of JIA according to disease manifestations within the first 6 months: oligoarticular JIA (o-JIA), rheumatoid factor negative (RF−) polyarticular JIA (p-JIA), RF-positive (RF+) p-JIA, enthesitis-related arthritis (ERA), systemic JIA (s-JIA), psoriatic arthritis and undifferentiated arthritis (1). JIA is the most common pediatric rheumatic disease with an incidence rate ranging from 1.6 to 23 cases per 100,000 people and a prevalence ranging from 3.8 to 400 cases per 100,000 people (2). JIA causes joint pain and swelling and limited range of joint motion and results in uveitis, osteopenia/osteoporosis, and growth retardation if it is not diagnosed early or treated promptly or effectively (2). However, the diagnosis of JIA relies mainly on clinical assessments and ruling out other pediatric diseases that manifest as chronic arthritis because of the lack of reliable diagnostic biomarkers. Since the development of new biological agents in the early 2000s, the outcomes of JIA in children have substantially improved (3). However, the disease has not yet been cured, further research is needed to explore the complex immunopathological process of JIA and identify new biomarkers and therapeutic targets.

JIA is thought to be associated with genetic, epigenetic, and environmental factors, but its etiology is not fully clear (3–5). Over the years, many studies have claimed that JIA is caused primarily by dysregulation of the adaptive immune system. However, as the understanding of genetics and immunology in JIA has improved, increasing evidence has shown that the innate immune system is also involved in the pathogenesis of JIA (5–8). Specifically, neutrophils have been implicated in the disordered immune response in JIA (6, 7). It is well evidenced that abundant activated neutrophils accumulate in the synovial fluid of inflamed joints in JIA patients (9, 10). In polyarticular JIA, peripheral blood neutrophils are chronically activated even when the patient is in clinical remission (6), and activated neutrophils can form neutrophil extracellular traps (NETs) (11–13), the process of NET formation, called NETosis, was originally thought to be a process of neutrophil death distinct from apoptosis and necrosis (14–16). Notably, excessive NET formation causes an inflammatory imbalance and dysregulation of adaptive immune responses by presenting major sources of autoantigens (17, 18) and danger-associated molecular patterns (DAMPs) (19, 20) and mediating complement and inflammasome activation (21–24). Additionally, NETs can activate T cells and autoreactive B cells, induce B-cell expansion, and promote B-cell and Th17 cell differentiation (25–28). Therefore, the role of NETs in autoimmune diseases is currently gaining increasing attention, and accumulating evidence suggests that NETs play a vital role in the initiation and perpetuation of autoimmune diseases, such as rheumatoid arthritis (RA), systemic lupus erythematosus (SLE) and anti-neutrophil cytoplasmic antibody (ANCA)-associated vasculitis (AAV) (17, 18, 20, 29). In animal models of antigen-induced arthritis, NETs contribute to articular pain and mediate joint edema, maintaining the inflammatory response (30).

JIA is not a single disease; this heterogeneity implies that different pathogenetic mechanisms underlie the various JIA subtypes. NET formation has been studied in o-JIA and p-JIA (31). In this study, NET formation was further studied in different subtypes of JIA in a larger size cohort. As such, we explored NET formation in patients with three subtypes of JIA, i.e., o-JIA, p-JIA, and ERA, respectively, and whether NET-derived products could be biomarkers for diagnosing JIA and monitoring disease activity. Furthermore, TNF-α plays a pivotal role in the pathogenesis of JIA (2, 7), and whether NETs are involved in TNF-α-mediated inflammation in JIA was investigated.

## Methods

### Human samples

Fifty-eight JIA patients who fulfilled the International League of Associations for Rheumatology (ILAR) criteria (1) were screened for inclusion in this study. Patients with other inflammatory or autoimmune diseases, metabolic diseases, and malignancies were excluded. Thirty healthy volunteer controls (HCs) who underwent physical examinations or elective surgery and had not received any medication for any disease were enrolled, and there was no significant difference in age and sex between HCs and patients. All participants were infection-free for at least one month and were recruited from Wuhan Children’s Hospital of Tongji Medical College, Huazhong University of Technology and Science between July 2022 and August 2023. Clinical and laboratory data and blood samples were collected. Fresh neutrophils were isolated from 5 mL of EDTA-anticoagulated peripheral blood by density centrifugation using Polymorphprep™ (Axis-Shield) according to the manufacturer’s protocol within 1-2 hours after collection. Contaminating erythrocytes were lysed with red blood lysis buffer (0.83% (w/v) for 4-5 min at room temperature. Trypan blue exclusion indicated that cell viability was ≥97%, and the purity of the isolated neutrophils was ≥ 90% according to the forward and side of scatter plots generated from the flow cytometric analyses. Neutrophils were further analyzed by flow cytometry (Attune NxT, AFC2, Thermo Fisher) after incubation with a fluorescein isothiocyanate (FITC)-conjugated anti-CD15 antibody (BioLegend, 394705). The purified neutrophils were resuspended in phenol red-free RPMI 1640 medium supplemented with 10% heat-inactivated fetal bovine serum (FBS) at 2.5×10^6^ cells/mL. The plasma samples were aliquoted and stored at −80°C until further analysis. This study was approved by the Wuhan Children’s Hospital Committee for Research Ethics and was performed following the Declaration of Helsinki. Written informed consent was obtained from all participants’ parents. Because of the limited amount of peripheral blood available, not all patients were included in each experiment.

The demographic characteristics of the patients are summarized in **Table 1**. The Juvenile Arthritis Disease Activity Score 27 (JADAS27) includes the following four measures: physician’s global assessment of disease activity, as measured on a 0–10 visual analog scale (VAS) where 0 = no activity and 10 = maximum activity; parent global assessment of well-being, as measured on a 0-10 VAS where 0 = very well and 10 = very poor; erythrocyte sedimentation rate (ESR), which was normalized to a 0 to 10 scale; and the number of joints with active disease (32, 33).

**Table 1:** Demographic, clinical, and laboratory characteristics.

Subtypes	o-JIA	p-JIA	ERA	All cases
**Number (%)**	29 (50%)	18 (31%)	11 (18.9%)	58 (100%)
**Gender (males, %)**	11 (37.93%)	4 (22.22%)	11 (100%)	26 (44.83%)
**Age (mean ± SEM, y)**	8.473 ± 0.7530	7.366 ± 1.151	10.68 ± 0.7825	8.548 ± 0.5524
**JADA S27 (mean ± SEM)**	12.27 ± 0.8272	15.11 ± 1.773	15.15 ± 1.512	13.91 ± 0.7342
**RF positivity (n, %)**	0 (0%)	7 (38.8%)	0 (0%)	7 (12.1%)
**HLA-B27 (n, %)**	0 (0%)	0 (0%)	4 (36.4%)	4 (6.9%)
**ANA positivity (n, %)**	13 (44.9%)	7 (38.9%)	7 (63.6%)	27 (45.55%)
**active uveitis (n)**	3 (10.35%)	0 (0%)	0 (0%)	3 (5.17%)
**ESR (median with IQR, mm/hour)**	6 (4-10)	8 (4-18)	11 (6-29.5)	7.5 (4-13)
**hs-CRP (median with IQR, mg/L)**	4.285 (2.035-13.63)	2.77 (1.44-31.55)	13.4 (6.105-69.95)	4.42 (1.6-16.7)
**Treatment**	12 (41.38%)	7(44.44%)	0 (0%)	19 (34.48%)
**GCs (n, %)**	1 (3.45%)	1 (5.56%)	0 (0%)	2 (5.17%)
**csDMARDs (n, %)**	12 (100%)	7 (100%)	0 (0%)	19 (100%)
**anti-TNF-a (n, %)**	10 (83.33%)	7 (100%)	0 (0%)	17 (90%)

o-JIA, oligoarticular juvenile idiopathic arthritis; p-JIA, polyarticular JIA; ERA, enthesitis-related arthritis; y, years; ANA, anti-nuclear antibodies; HLA-B27, human leukocyte antigen B27; RF, rheumatoid factor; ESR, erythrocyte sedimentation rate; hs-CRP, hypersensitive C-reactive protein; IQR, interquartile ranges; JADAS27, Juvenile Arthritis Disease Activity Score 27; GCs, glucocorticoids; csDMARDS, conventional synthetic disease-modifying antirheumatic drugs; anti-TNF-a, anti-tumor necrosis factor-alpha antibody.

### *In vitro* stimulation and inhibition studies

Freshly isolated neutrophils (2.5×10^5^ cells, 100 µL) from JIA patients or HCs were seeded in 24-well plates on 0.001% poly-L-lysine-coated glass coverslips and incubated for 30-40 min at 37°C in 5% CO_2_. Then, the neutrophils were incubated with phorbol-12-myristate-13-acetate (PMA, 30 nM) (P1585-1MG, Sigma) for 3 hours or recombinant human TNF-α (100 ng/mL) (570102, BioLegend) for 6 hours at 37°C in 5% CO_2_. In addition, neutrophils were cultured without any stimulus for 6 hours to assess spontaneous NET formation. To visualize whether TNF-α-induced NET formation was inhibited by TNF-α antagonists, neutrophils were seeded in the same way as described above and pretreated with a humanized anti-TNF-α antibody (adalimumab, Humira, Abbvie) (4 μg/mL, equivalent to the average serum concentration in the human body) for 15 minutes prior to TNF-α stimulation. The concentrations and time points for neutrophil stimulation were determined based on the optimized protocol.

### Visualizing NET formation via immunofluorescence confocal microscopy

After treatment as described above, the neutrophils were immediately fixed with 4% paraformaldehyde, rinsed three times with PBS, and blocked with 3% BSA. For immunofluorescence labeling, the neutrophils were stained using a rabbit anti-myeloperoxidase (MPO) mAb (1:800) (ab208670, Abcam) overnight at 4°C and then with an Alexa Fluor 488-conjugated goat anti-rabbit secondary antibody (A-11008; Thermo Fisher) for 1 hour at room temperature. The DNA was counterstained with 4′,6-diamidino-2-phenylindole (DAPI) (C1005, Beyotime) for 5 minutes. Neutrophils and NETs were visualized using a Nikon Eclipse-Ti-S fluorescence microscope (Nikon, Tokyo, Japan). Five randomly chosen fields of each coverslip were observed, and fluorescence images of MPO and DNA were analyzed using ImageJ software (National Institutes of Health, NIH). The results are expressed as the percentage area of the microscopic field of view occupied by NETs under a 20× objective.

Since NETs are fragile and easily shed during surgery, we used SYTOX Green (Invitrogen Life Technologies, San Diego, CA, USA) to stain DNA to further visualize NET formation. The cells were fixed with 4% paraformaldehyde and rinsed 3 times with PBS. DNA was stained with 0.2 mM SYTOX Green dye for 15 min, and NETs were visualized using a Nikon Eclipse-Ti-S fluorescence microscope. A total of 5 randomly selected fields from different regions of each coverslip were imaged with a 20× objective.

### Quantification of NET release via a fluorescence microplate assay

Fluorescence microscopy is laborious and time-consuming and is not suitable for the assessment of a large number of samples. Hence, a fluorescence microplate assay was used to determine the amounts of NET formation. Freshly isolated neutrophils (4×10^4^ cells per well) were cultured in 96-well black microtiter plates in RPMI 1640 medium supplemented with 10% FBS containing 0.2 µM SYTOX Green and stimulated with PMA (30 nM) or with TNF-α (100 ng/mL) for 6 hours. Neutrophils were also incubated without any stimulus for 6 hours to measure spontaneous NETosis. To quantify the inhibitory effects of adalimumab and DPI on TNF-α-induced NET formation *in vitro*, neutrophils were pretreated with adalimumab (4 μg/mL) or DPI (10 μM) (diphenyl iodide, NADPH oxidase inhibitor, Enzo Life Sciences) for 15 minutes prior to the addition of TNF-α. The samples were analyzed in triplicate. After incubation, the absorbance of each well of the 96-well black microtiter plates was measured with an EnSpire Multimode Plate Reader (PerkinElmer, Inc) at 485 nm (excitation)/520 nm (emission). The data are expressed in relative fluorescence units (RFUs) and were calculated by subtracting the fluorescence intensity of the control cells at time 0.

### Measurement of reactive oxygen species production

Neutrophils (1.5×10^5^ cells) were incubated with TNF-α (100 ng/mL) for 3 hours at 37°C or were not incubated. Then, CellROX™ Green (C10444, Invitrogen) was added to the cells, which were then incubated at 37°C for 15 minutes. Then the cells were washed with PBS to remove the unbound dye, labeled with 7-AAD (559925, BD Pharmingen™), resuspended in PBS, and subsequently detected by flow cytometer (AttuneNxt, Thermo Fisher). The fluorescence intensity of individual cells was analyzed by FlowJo software.

### Western blot analysis

Neutrophils (2.5×10^6^/mL) isolated from HCs or JIA patients were either cultured in 24-well plates in the presence or absence of 100 ng/mL TNF-α (570102, BioLegend) for 3 hours at 37°C or were not cultured. Then, the cells were lysed with RIPA buffer (P0013B, Beyotime) containing a protease inhibitor cocktail (G2006, Servicebio), NaF (1 M, G2007-1, Servicebio), and Na_3_VO_3_ (100 mM, G2007-1, Servicebio). The cell lysates were separated by SDS−PAGE and electrotransferred onto nitrocellulose membranes (Amersham™ Protran™ 0.2 μm NC). The following primary antibodies were used: monoclonal anti-MPO (1:1000; Abcam ab208670), anti-GAPDH (1:1000, Cat No. CL594-60004, Proteintech), anti-phospho-PI3K (1:1000; 4292S, Cell Signaling Technology), anti-phospho-AKT (1:1000; Cell Signaling Technology, 4060 L), anti-phospho-extracellular signal-regulated kinase 1/2 (ERK1/2) (Abmart T40072) and anti-phospho-MAPK-CDK (mitogen-activated protein kinase/cyclin-dependent kinase) (1:1000; Cell Signaling Technology, 2325) antibodies. The secondary antibodies used were horseradish peroxidase (HRP)-conjugated goat anti-mouse immunoglobulin G (IgG) (1:10000) (Proteintech, SA00001-1) and HRP-conjugated goat anti-rabbit IgG (1:8000) (Proteintech, SA00001-2). The bound antibodies were detected using an enhanced chemiluminescence system.

### Quantification of cell-free DNA concentrations

Cell-free DNA concentrations were quantified using a Helixyte™ Green dsDNA Quantitation Assay Kit (AAT Bioquest Catalog number: 17650). HC and JIA patient plasma samples were diluted 1:10 and incubated with Helixyte Green™ working solution for 5-10 min at room temperature away from light. Then, the fluorescence intensity was measured with an EnSpire Multimode Plate Reader (Ex/Em = 490/525 nm). The data were analyzed using serial dilutions of calf thymus DNA to generate a calibration curve.

### Analysis of MPO-DNA complex levels by ELISA

A novel capture ELISA was used to quantify NET levels in JIA patients and controls by measuring the levels of the MPO-DNA complex in human plasma. The wells of 96-well plates were coated with 5 μg/mL mouse anti-human myeloperoxidase antibody (clone D02-2A1, Bio-Rad) (100 μL per well) as the capture antibody overnight at 4°C. After the wells were blocked in 1% BSA (150 μL per well) and washed 3 times with 0.05% PBST (300 μL each), 20 μL of patient plasma combined with 80 μL of incubation buffer containing a peroxidase-labeled anti-DNA mAb (Cell Death ELISA Plus, Roche; Cat. No: 11774425001) was added to each well according to the manufacturer’s instructions. After three hours of incubation at RT on a shaker (300 rpm), the plates were washed with 0.05% PBST (300 μL each), and 100 μL of the peroxidase substrate (ABTS) from the kit (Cell Death Detection ELISA^PLUS^, Roche, Cat. No: 11774425001) was added. The absorbance at a measurement wavelength of 405 nm and a reference wavelength of 490 nm was measured with a BioTek Synergy H1 microplate reader after 40 minutes of incubation at room temperature in the dark. The difference between the measured and reference absorbance was calculated as MPO-DNA complex level in the plasma of JIA patients and HCs.

### Statistical analysis

Statistical analysis was performed using SPSS 18.0 statistical software or GraphPad Prism software version 8.0. Continuous variables are presented as the means ± standard errors of the means (SEMs) or medians and interquartile ranges (IQRs). For continuous variables with normal distributions, Student’s t-tests or paired-sample t tests were used to analyze differences. For nonnormally distributed data, the Mann−Whitney U test or Wilcoxon test was used for comparisons between two groups. One-way ANOVA or the Kruskal−Wallis test was used for comparisons among multiple groups. Spearman’s or Pearson’s rank correlation analyses were used as indicated to evaluate associations between the levels of NET components and clinical parameters, and multiple linear regression analysis was used for multivariate analysis. Receiver operating characteristic (ROC) curves were generated and the area under the curve (AUC) was analyzed to measure the sensitivity and specificity of NET-derived products for the diagnosis of JIA. *P* values less than 0.05 were considered to indicate statistical significance (*p<0.05, **p<0.01, ***p<0.001, ****p<0.0001).

## Results

### Detailed demographic, clinical, and laboratory characteristics of JIA patients

Fifty-eight patients with JIA participated in the study: 29 patients with o-JIA, 18 patients with p-JIA, and 11 patients with ERA. o-JIA was the most common subtype, accounting for 50% of all JIA cases. The percentages of patients with p-JIA and ERA are also shown in **Table 1**. The population consisted of 32 female and 26 male children. The mean age was 8.548 ± 0.5524 y (95% CI 7.442-9.655). The mean JADAS27 was 13.91 ± 0.7342 (95% CI 12.44-15.38). The median ESR was 7.5 mm/h (IQR 4-13; range 2 and 51 mm/h). Thirty-nine patients with JIA did not receive medical treatment, nineteen patients had already undergone treatment for six months, and seventeen patients had received TNF-α inhibitor therapy. Thirty age- and sex-matched HCs were recruited as controls.

### Excessive NET formation in neutrophils from patients with JIA spontaneously or in response to PMA and TNF-α *in vitro*

**Figure 1:**
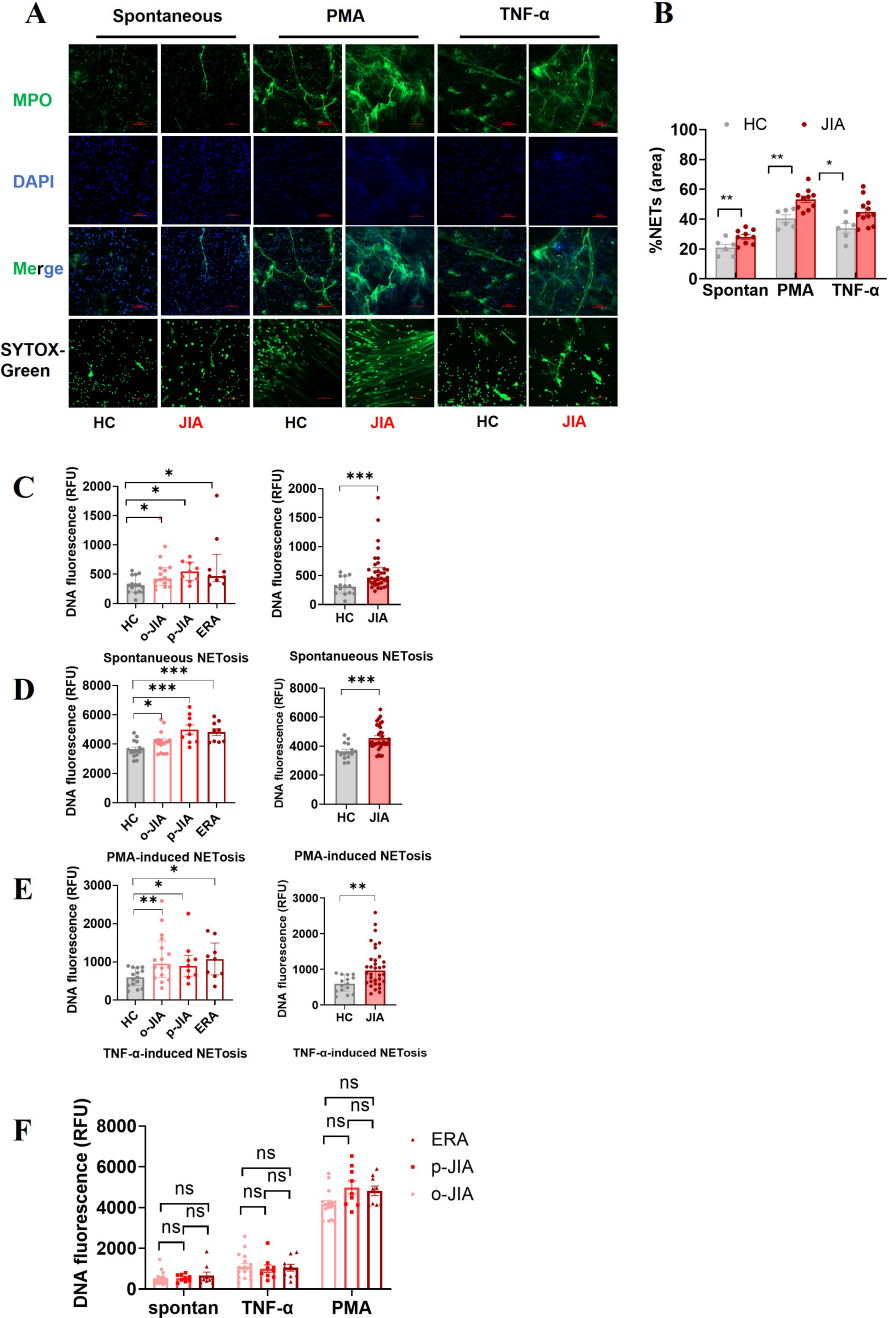
Neutrophils derived from the peripheral blood of JIA patients increase NET formation *in vitro*, either spontaneously or in response to TNF-α or PMA. **(A)** Representative images of NETs released by neutrophils derived from at least 10 patients with JIA and 6 HCs spontaneously or in response to PMA or TNF-α. NETs were double immunostained for extracellular DNA (DAPI; blue) and MPO (MPO antibody; green), and NETs were identified by the presence of extracellular DNA stained with SYTOX-Green. Images were captured at 20× magnification; the scale bars represent 100 µm. **(B)** In a quantitative analysis of the above experiments, the percentages of NET-occupied areas in the total area in the JIA group were markedly higher than those in the HC group. **(C–F)** Quantitative assessment of NET formation in the o-JIA, p-JIA, and ERA groups by microplate assays. Neutrophils isolated from patients with o-JIA, p-JIA, and ERA and healthy controls were incubated for 6 hours under the conditions of **(C)** no intervention, **(D)** PMA (30 nM) stimulus, or **(E)** the addition of TNF-a (100 ng/ml). **(F)** The amounts of NETs were compared among the three subtypes of o-JIA, p-JIA, and ERA, respectively. The results are expressed as the fluorescence intensity of DNA in NETs. One-way ANOVA or the Kruskal−Wallis test was used to compare three or more groups. Student’s independent-sample t-test was used to compare the two groups. The bar graphs show the mean ± SEM or median with IQR. *p<0.05, **p<0.01, ***p<0.001, ns, nonsignificant; spontan, spontaneous.

MPO is embedded in extracellular DNA which is the major backbone of NETs (13). NETs were identified by the colocalization of extracellular DNA and MPO, and the amounts of NETs were quantified by fluorescence microscopy and a fluorescence microplate assay (**Figure 1**). As shown in **Figures 1A, B**, the percentages of the NET-occupied area were markedly greater in JIA-derived neutrophils than in HC-derived neutrophils after no stimulation (28.75 ± 1.355% vs. 21 ± 2.236%, *p* = 0.0065), PMA stimulation (53.4 ± 2.172% vs. 40.5 ± 2.527%, *p* = 0.0021) or TNF-α stimulation (44.83 ± 2.628% vs. 34 ± 3.215%, *p* = 0.0247).

A fluorescence microplate assay was utilized to further quantify the levels of NETs in patients with different subtypes of JIA, including o-JIA, p-JIA, and ERA. Our findings showed that there were more NETs formed by neutrophils derived from 16 o-JIA patients, 9 p-JIA patients and 9 ERA patients than from 15 HCs with no stimulation (*p* = 0.0436, *p* = 0.0119, and *p* = 0.0193, respectively; **Figure 1C**), PMA stimulation (*p* = 0.0376, *p* = 0.0003, and *p* = 0.0002, respectively; **Figure 1D**) or the addition of TNF-a (*p* = 0.0059, *p* = 0.0378, and *p* = 0.0164, respectively; **Figure 1E**). Moreover, there were significant differences in the amounts of NETs released by neutrophils after the indicated intervention between in entire cohort of JIA patients and in HCs (*p* = 0.0005, *p* = 0.0004 and *p* = 0.0014, respectively; **Figures 1C–E**), which was consistent with the results of fluorescence microscopy. Importantly, our findings revealed no significant differences in NET formation among o-JIA, p-JIA and ERA patients (*p* > 0.05, **Figure 1F**). Overall, neutrophils derived from patients with the three subtypes of JIA were more prone to release NETs in the presence or absence of TNF-α or PMA stimulation and further *in vitro* verification of TNF-α mediated NET generation was performed.

### Increased plasma levels of NET-derived products in JIA patients: potential biomarkers for diagnosis and disease activity

**Figure 2:**
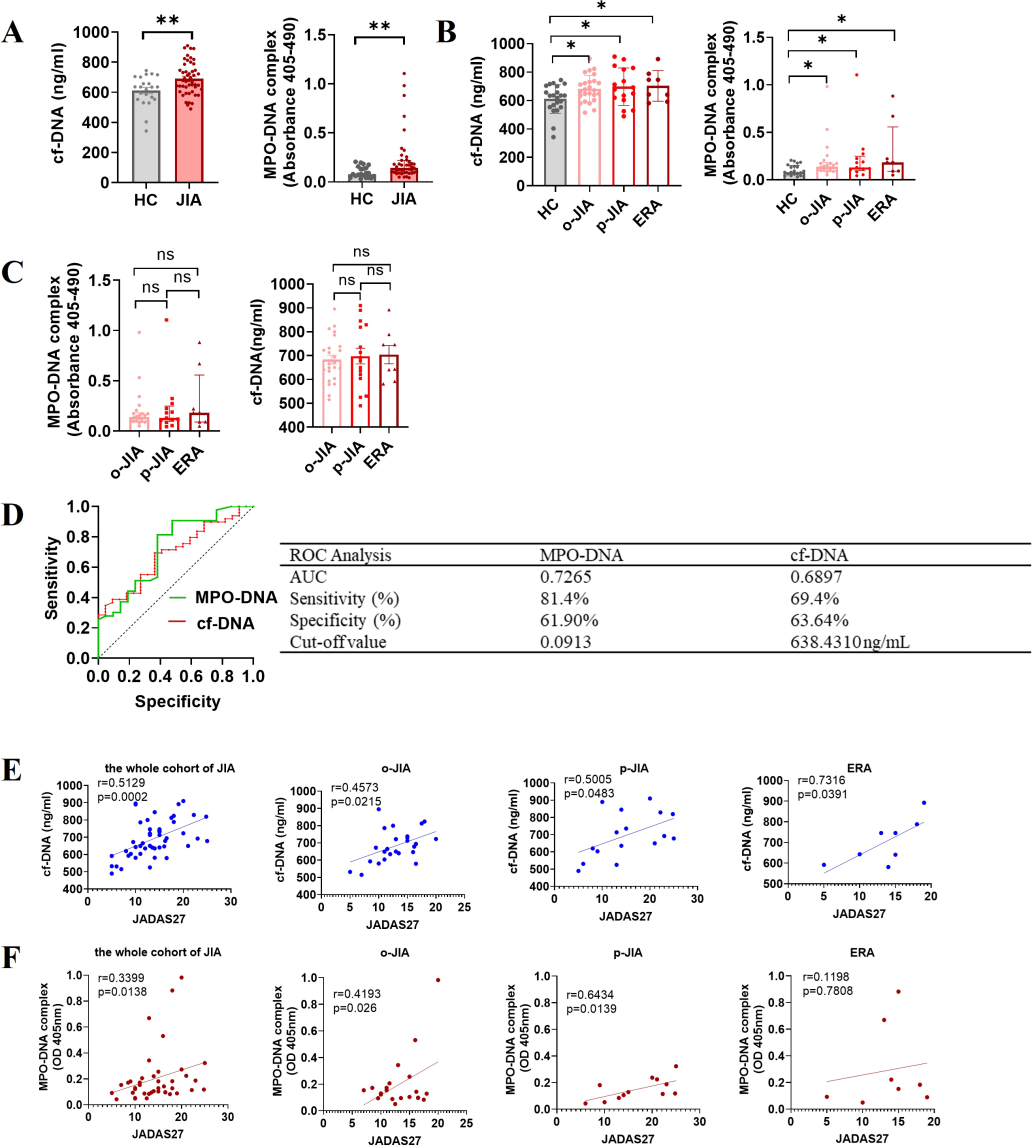
Excessive NET formation exists in the peripheral circulation of patients with JIA, with elevated NET-derived components as biomarkers for diagnosis and disease activity. **(A)** The plasma levels of NETosis-derived products, cf-DNA and MPO-DNA complexes, were compared between JIA patients and HCs. **(B)** The plasma concentrations of cf-DNA in children with o-JIA (n = 25), p-JIA (n=16) and ERA (n = 8) were higher than those in HCs (n = 22). Meanwhile, there was an increase in the plasma levels of MPO-DNA complexes in 22 o-JIA patients, 13 p-JIA patients and 8 ERA patients compared to HCs (n = 21). The bar graphs show the mean ± SEM or the median with IQR. *p<0.05, **p<0.01. **(C)** The plasma levels of cf-DNA and MPO-DNA complexes were similar among the three subtypes of JIA. ns, nonsignificant. **(D)** ROC curve analysis of cf-DNA and MPO-DNA complexes to evaluate the accuracy of these parameters as diagnostic biomarkers of disease in JIA patients (AUC = 0.6897, *p* = 0.0110; and AUC = 0.7265, *p* = 0.0035, respectively). AUC, area under the curve. **(E)** The concentrations of cf-DNA were strongly and positively associated with the JADAS27 in the whole cohort of children with JIA (n = 49) and the three JIA subtypes, i.e., o-JIA (n=25), p-JIA (n = 16), and ERA (n = 8). **(F)** A significant positive correlation between the MPO-DNA complex levels and the JADAS27 in 42 patients with JIA, 22 patients with o-JIA, and 12 patients with p-JIA, but not 8 patients with ERA. R values of Spearman or Pearson’s rank correlation and *p* values of their null hypothesis are shown.

To quantify NET formation in the peripheral blood of JIA patients, plasma levels of cf-DNA and MPO-DNA complexes, as major components of NETs, were measured, there was a marked increase in levels of cf-DNA and MPO-DNA complexes in peripheral blood of JIA patients when compared to HCs [691.4 ± 15.37 ng/mL vs. 611.2 ± 21.53 ng/mL, *p* = 0.0043; 0.1430 (IQR:0.0952-0.2210) vs. 0.0797 (IQR: 0.05125-0.1500), *p* = 0.0030; **Figure 2A**]. Significantly, the plasma concentrations of cf-DNA in patients with o-JIA, p-JIA and ERA were significantly increased as compared to those in HCs (683.3 ± 18.73 vs. 611.2 ± 21.53 ng/mL, *p* = 0.0243; 697.9 ± 32.92 vs. 611.2 ± 21.53 ng/mL, *p* = 0.0162; 703.7 ± 38.14 vs. 611.2 ± 21.53 ng/mL, *p* = 0.0401, respectively; **Figure 2B**). Similarly, the plasma levels of MPO-DNA complex in patients with o-JIA [0.1356 (IQR: 0.0961-0.1806)], p-JIA [0.1295 (IQR: 0.09540-0.0.2485)] and ERA [0.1823 (IQR:0.08975-0.5570)] were higher than those in HCs [0.0797 (IQR:0.05125-0.1500)] (*p =* 0.0163, *p =* 0.0302 and *p =* 0.0258, respectively; **Figure 2B**), these results verified that excessive NET formation were present in peripheral blood of JIA patients. However, there were no differences in cf-DNA and MPO-DNA complex levels among the three JIA subtypes (*p* > 0.05, **Figure 2C**).

Significantly, ROC curve analysis showed that plasma levels of cf-DNA and MPO-DNA complex, as diagnostic biomarkers, could accurately distinguish JIA patients from HCs, with cut-off values of 638.4310 ng/mL and 0.0913, respectively (**Figure 2D**). **Figure 2E** showed that the plasma concentration of cf-DNA was strongly correlated with the JADAS27 in the whole cohort of JIA patients (r = 0.5129, *p* = 0.0002), and in patients with o-JIA, p-JIA, and ERA (r = 0.4573, *p* = 0.0215; r = 0.5005, *p* = 0.0483; and r = 0.7316, *p* = 0.0391, respectively). As shown in **Figure 2F**, a significant positive correlation between MPO-DNA complex levels and disease activity (JADAS27) was identified in the whole cohort of JIA patients (r = 0.3399, *p* = 0.0138) and even in o-JIA patients (r = 0.4193, p = 0.0260), and p-JIA patients (r = 0.6434, *p* = 0.0139), despite no significant correlation in ERA patients (r = 0.1198, *p* = 0.7808). Taken together, NETosis-derived products may be used to monitor disease activity, and thus predict the flares.

### Associations of immune-related laboratory parameters with cell-free DNA concentrations in JIA patients

**Figure 3:**
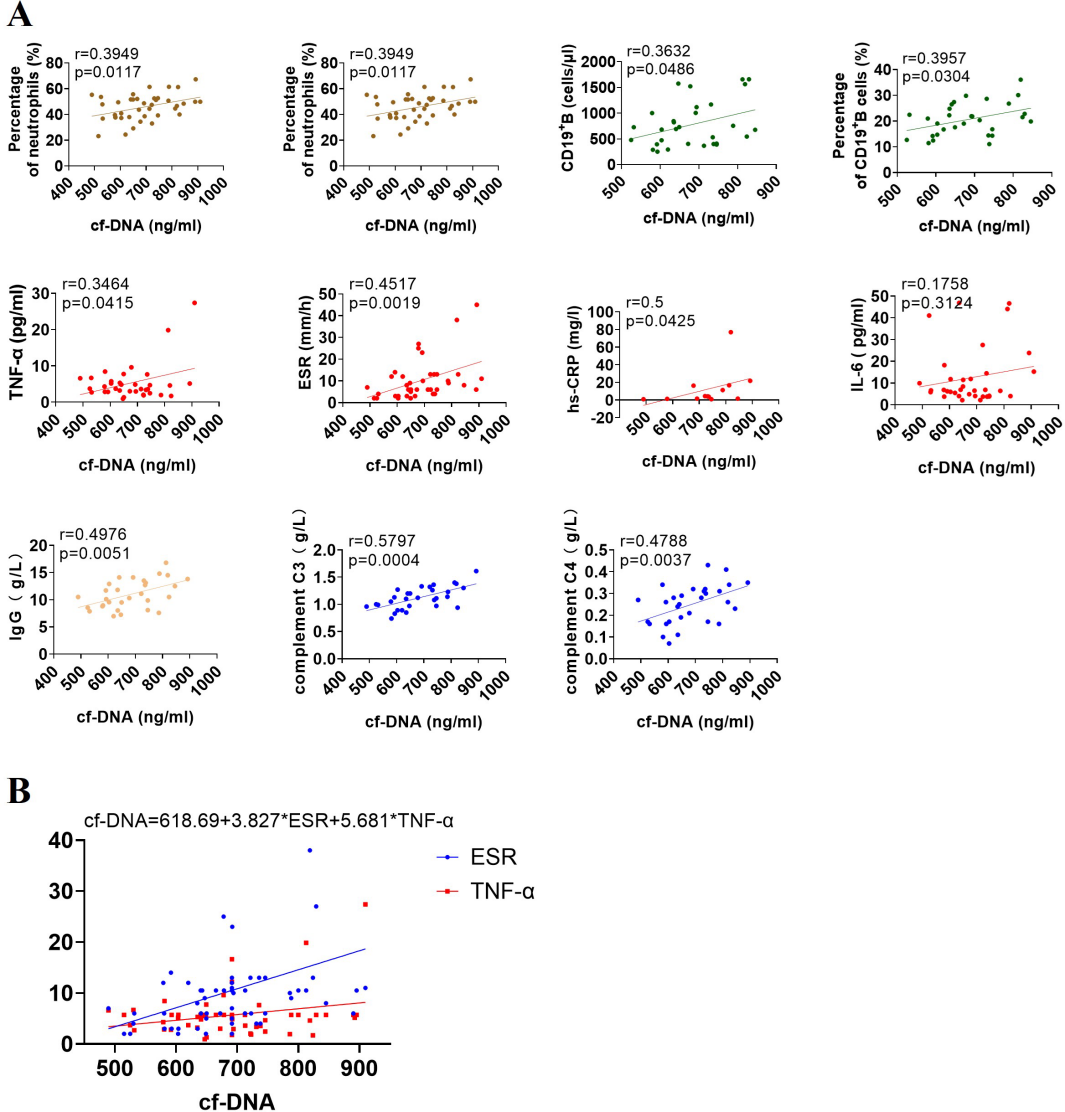
Associations of cf-DNA levels with immune-related parameters in the entire cohort of patients with JIA. **(A)** The concentrations of cf-DNA were strongly correlated with the number and percentage of neutrophils and CD19+ B cells, the expression of inflammatory markers (TNF-α, ESR, and hs-CRP), the levels of IgG, complement C3 and C4 in peripheral blood, but no correlation with IL-6 level. R values of Spearman or Pearson’s rank correlation and *p* values of their null hypothesis are shown. **(B)** ESR and TNF-α are independent variables by the multiple linear regression analysis.

Emerging research suggests that NET remnants can interact with immune components and immune cells to modulate immune response (20). In this study, correlation studies demonstrated that the plasma concentrations of cf-DNA strongly and positively correlated with some immune-related parameters, such as the number and percentage of neutrophils and B cells, hs-CRP, ESR, and TNF-α levels, total IgG production and complement C3 and C4 levels in peripheral circulation, whereas nonsignificant correlation between IL-6 level and cf-DNA concentration was observed (**Figure 3A**). Subsequently, the 10 statistically significant variables were analyzed by multiple linear regression analysis, and the multiple linear regression equation established was statistically significant (*P* < 0.05; **Figure 3B**), only ESR and TNF-α levels were positively associated with cf-DNA concentration and were found to be independent variables, implying that TNF-α is a major inducer in triggering NET formation in JIA patients, and further verifying that cf-DNA concentration could be a biomarker for monitoring disease activity.

### TNF-α inhibitors suppress TNF-α-induced NET formation both *in vitro* and *in vivo*

**Figure 4:**
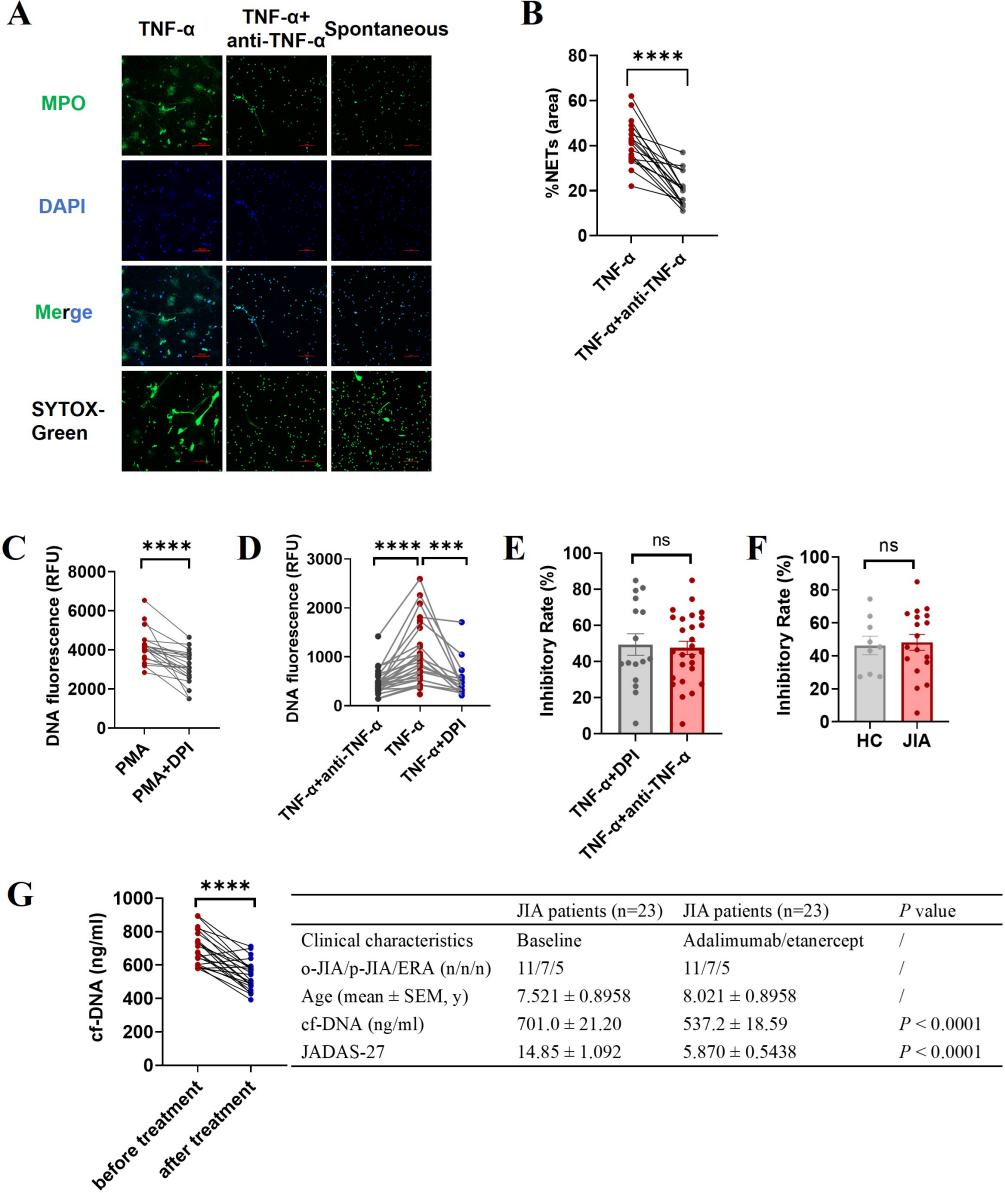
Effect of an anti-TNF-a antibody (adalimumab) on NET formation. Neutrophils isolated from JIA patients and HCs were pretreated with adalimumab (4 μg/ml) prior to the addition of TNF-α. **(A)** Representative images of TNF-α induced NETs and minimal NET formation after adalimumab intervention. The third column is an image of spontaneous NET formation as a negative control. **(B)** The percentage of the NET-occupied area under TNF-α stimulation was significantly decreased in the presence of adalimumab (n=18, 41.22% ± 2.351 vs. 20.39% ± 1.683, *p* < 0.0001). **(C–F)** The levels of NET release were measured via microplate assays to further evidence the ability of adalimumab to inhibit NET generation. **(C)** DPI can effectively inhibit PMA-induced NETosis. **(D)** Adalimumab (n=27) and DPI (n=16) effectively inhibited TNF-α-induced NET formation (n=27). **(E)** Comparison of the inhibitory effects of adalimumab and DPI on TNF-α-induced NET formation. **(F)** The inhibitory rate of adalimumab in NETosis by neutrophils from JIA (n=18) and HCs (n=9) under TNF-α stimulation. **(G)** The effect of TNF-α inhibitors on cf-DNA concentration and JADAS27 in JIA patients. The data were analyzed with paired-sample t test or unpaired-sample t test. The bar graphs show the mean ± SEM. ***p<0.001, ****p<0.0001, ns, nonsignificant.

*In vitro* inhibition study showed that adalimumab (a humanized anti-TNF-α antibody) effectively suppressed TNF-α-induced NET formation, as assessed by fluorescence microscopy (*p* < 0.0001; **Figures 4A, B**). NADPH oxidase inhibitor (DPI) can almost completely inhibit PMA-induced NETosis (14, 34) which was further verified in this study (n=21, *p* < 0.0001; **Figure 4C**), and was used as a positive control of effective inhibition of NETosis. Our results showed that DPI could inhibit TNF-α-induced NET formation (n = 16 *p* = 0.0008; **Figure 4D**), and the inhibitory effects of adalimumab and DPI on suppressing TNF-α-induced NETosis were similar (47.57% ± 3.665 vs. 49.41% ± 23.79, *p* = 0.7821; **Figure 4E**). Moreover, there was no difference in the inhibitory effects of adalimumab in suppressing TNF-α-induced NETosis between JIA patients and healthy controls (*p* = 0.8160; **Figure 4F**).

Next, we examined whether TNF-α inhibitors could prevent NETosis *in vivo*. As shown in **Figure 4G** that patients with JIA had a decreased plasma concentration of cf-DNA after six months of treatment with TNF-α inhibitors (adalimumab or etanercept), along with significantly improving disease activity (reduced JADAS27) (n = 23, *p* < 0.0001), indicating that TNF-α inhibitors can effectively inhibit NET formation *in vivo*.

### High expression of NET-associated signaling components in JIA-derived neutrophils

**Figure 5:**
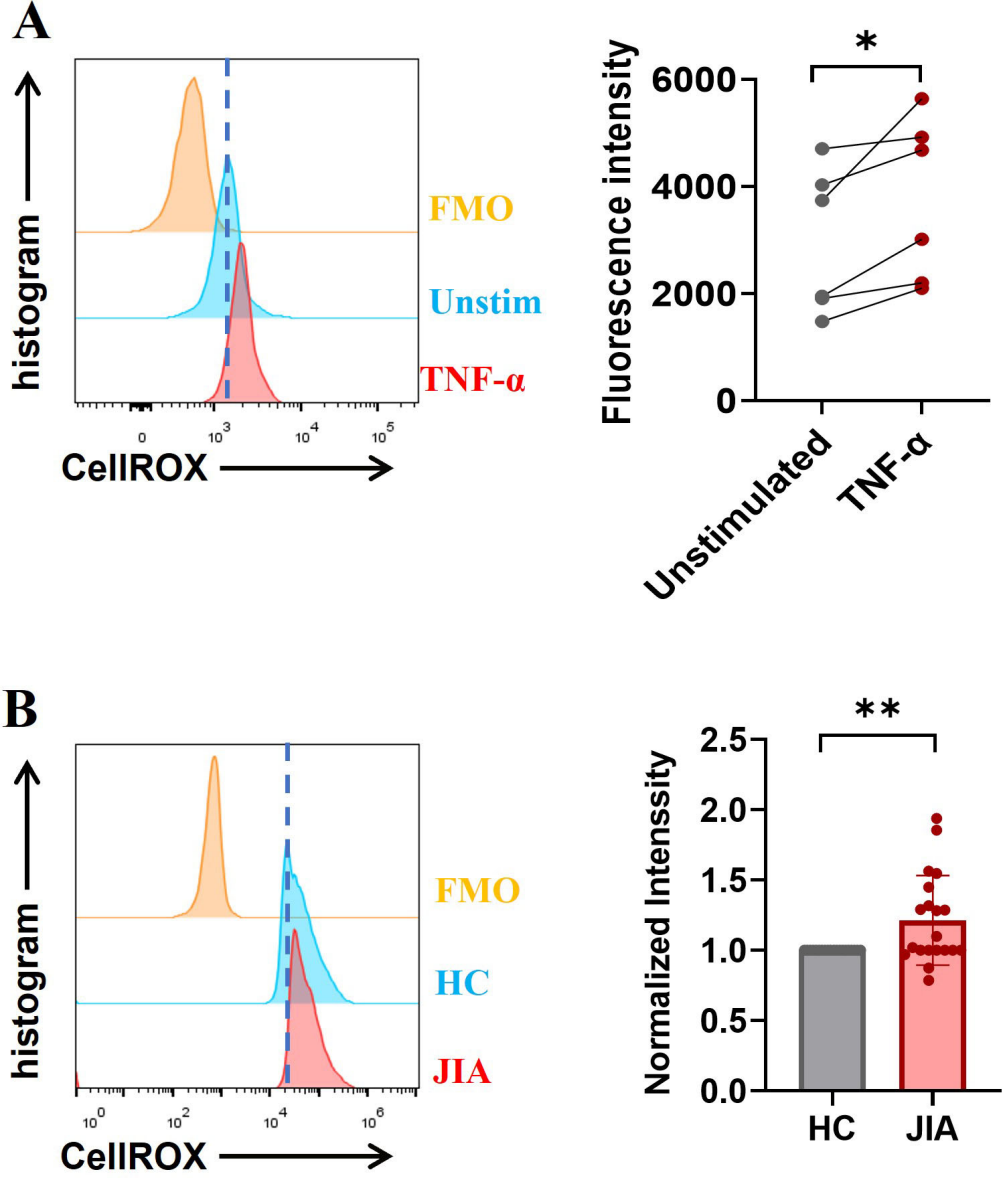
ROS levels were measured ex vivo using a FACSCalibur flow cytometer. **(A)** The exposure of neutrophils to TNF-α increased in the production of ROS. **(B)** Baseline ROS levels in neutrophils derived from JIA patients were higher than those in HC-derived neutrophils (n = 20; *p* = 0.0074). The paired-sample t test was used to analyze all the data. * *p*< 0.05, **p<0.01.

**Figure 6:**
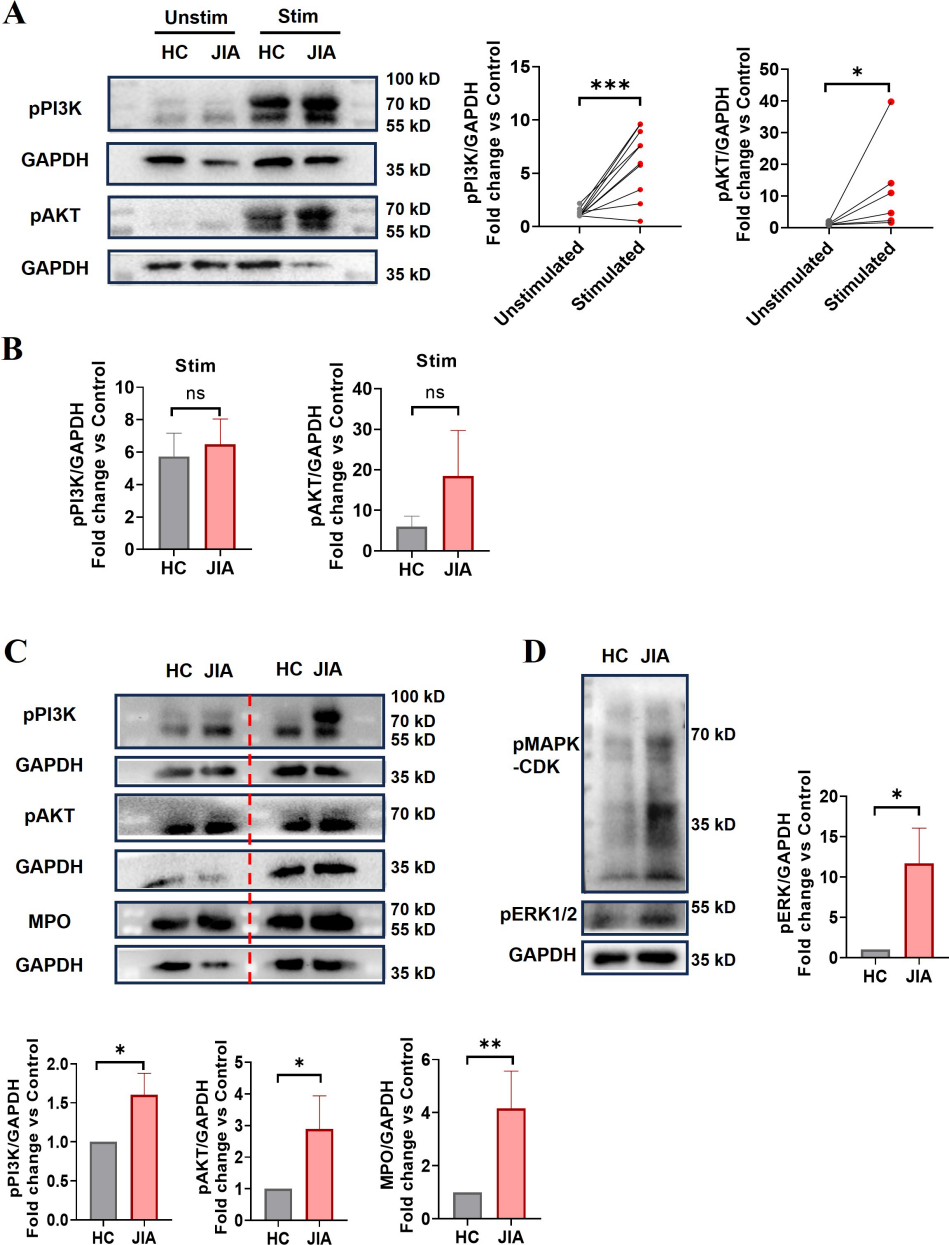
The expression levels of NET-associated signaling components. **(A)** The phosphorylation of PI3K and AKT in neutrophils purified from JIA patients and HCs before and after TNF-α stimulation (p-PI3K n = 10, *p* = 0.0008; p-AKT, n = 6, *p* = 0.0313). The paired-sample t test and Wilcoxon test were used to analyze the data. **(B)** Comparison of phosphorylated PI3K and AKT protein levels in neutrophils derived from JIA patients and HCs after TNF-α stimulation (n = 5, *p* = 0.7340; n = 3, *p* = 0.3386, respectively). **(C)** Phosphorylation of PI3K (15 HCs vs. 18 JIA patients, *p* = 0.0222) and AKT (n = 10 *p* = 0.0135) and the protein expression of MPO (n = 15, *p* = 0.0012) in neutrophils derived from JIA patients and HCs. **(D)** MAPK-CDK and ERK1/2 phosphorylation was assessed (6 HCs vs. 7 JIA patients *p* = 0.0239). **(A, C, D)** Representative western blots. The Mann−Whitney U test was used to compare two groups. Stim, stimulated; unstim, unstimulated. The bar graphs show the mean ± SEM. *p < 0.05, **p < 0.01, ***p < 0.001, ns, nonsignificant.

NADPH oxidase, a key enzyme in redox signaling, is a major generator of reactive oxygen species (ROS) *in vivo*. NADPH oxidase-mediated ROS generation is involved in most of the mechanisms underlying NETosis (35). TNF-α-induced NET formation was suppressed by DPI (**Figure 4E**), and ROS production in neutrophils stimulated with TNF-α was significantly increased (n = 6, *p* = 0.0266; **Figure 5A**), indicating that NADPH oxidase-mediated ROS production was an important factor driving TNF-α-induced NET formation. To further clarify the upstream signals mediating NADPH oxidase activation, the effect of TNF-α on phosphoinositide 3-kinase (PI3K)-AKT signaling activation was examined, because this signaling pathway is involved in ROS production by NADPH oxidase (36, 37). Western blotting revealed that the levels of phosphorylated PI3K and AKT in neutrophils from JIA patients and HCs were elevated after TNF-α stimulation (**Figure 6A**), especially in JIA-derived neutrophils, despite nonsignificant differences between patients and HCs (**Figure 6B**). These results suggested that TNF-α induced NETosis through ROS- and PI3K-Akt-dependent signaling pathways.

Excessive NET formation existed in peripheral circulation of patients with o-JIA, p-JIA and ERA, so it is urgent to explore the molecular mechanisms of NETosis in JIA patients. Our findings demonstrated that basal ROS production, MPO expression, and phosphorylated PI3K and AKT levels in JIA-derived neutrophils were markedly increased compared with those in HC-derived neutrophils (**Figures 5B**, **6C**). Previous studies have shown that the MAPK-ERK1/2 signaling pathway is involved in NET formation through the activation of NADPH oxidase (38, 39), and NET formation is controlled by the activation of cyclin-dependent kinases 4 and 6 (CDK4/6) (40). As shown in **Figure 6D**, the levels of MAPK-CDKs and ERK1/2 phosphorylation were markedly higher in JIA-derived neutrophils than in HC-derived neutrophils. Overall, there might be distinct activated NETosis-associated signaling pathways in the hyperinflammatory milieu of JIA.

## Discussion

Recent research has highlighted that NETs are released by neutrophils upon activation and play central roles in the initiation and perpetuation of inflammation and autoimmune responses (11), and it has been verified that neutrophils are activated in JIA patients (6, 9). In this study, the capacity of neutrophils purified from patients with o-JIA, p-JIA, and ERA to generate NETs spontaneously and in response to TNF-α or PMA *in vitro* was significantly higher than that of cells derived from HCs (**Figure 1**). Importantly, patients with JIA had elevated plasma levels of cf-DNA and MPO-DNA complexes which were identified as NETosis-derived products compared to healthy controls (**Figures 2A, B**). Strangely, there was no significant difference in NET formation both *in vivo* and ex vivo among the three subtypes of JIA i.e., o-JIA, p-JIA, and ERA (**Figures 1F**, **2C**). Therefore, we concluded that these activated neutrophils in JIA patients most likely contribute to the pathogenesis of JIA by releasing NETs.

ROC curve analysis demonstrated that circulating levels of cf-DNA and MPO-DNA complexes could serve as potential diagnostic biomarkers for accurately distinguishing JIA patients from HCs (**Figure 2D**). In addition, although there was no significant correlation between the MPO-DNA complex levels and the JADAS27 in ERA patients, this was most likely due to the small sample size (n = 8), the plasma levels of MPO-DNA complex were associated with the JADAS27 in o-JIA and p-JIA patients (**Figure 2F**), and cf-DNA plasma concentrations were strongly and positively associated with the JADAS27 in patients with o-JIA, p-JIA and ERA (**Figure 2E**). We believed that plasma levels of NET-derived products in patients with JIA could be sufficient for monitoring disease activity, and could be used to predict disease flares. Recent research has also shown that NET formation is increased in o-JIA patients and is correlated with the disease activity score (cJADAS10), but not in p-JIA patients (31), which are not entirely consistent with our conclusions. The main reason is likely due to their small sample size (o-JIA, n = 4 and p-JIA, n = 3). Our research not only examined a large sample size, but also investigated NET formation via *in vitro* stimulation studies and in the peripheral circulation of patients with o-JIA, p-JIA and ERA using a variety of methods, meaning that our study is more reliable than the previous data. In addition, there is rarely a study investigating NET formation in patients with JIA, we systematically explored NETosis in patients with the three subtypes of JIA i.e., o-JIA, p-JIA, and ERA, respectively.

Ayako Ohyama et al. have evidenced that there was significant CitH3 overexpression in pGIA (peptide glucose-6-phosphate isomerase-induced arthritis) joints, and anti-IL-6 receptor antibodies could decrease neutrophilic infiltration and NETosis in the joints of pGIA (41). In addition, IL-6 inhibitors are highly effective and have been approved for use in systemic JIA (2). However, correlation analysis revealed that cf-DNA concentration and IL-6 levels did not significantly correlate in JIA patients consisted of o-JIA, p-JIA and ERA patients, indicating IL-6 may not play a major role in o-JIA, p-JIA and ERA. Simple linear regression of correlation studies showed that the number and percentage of neutrophils and CD19+ B cells, the levels of the systemic inflammatory markers TNF-α, hs-CRP and ESR, the serum IgG and complement C3 and C4 levels were strongly and positively correlated with cf-DNA levels (**Figure 3A**). However, only ESR and TNF-α levels are positively associated with cf-DNA concentration and are independent variables by multiple linear regression analysis (**Figure 3B**). TNF-a has been identified as a proinflammatory cytokine with a central role in arthritis, which was further proved by the clinical efficacy of the TNF-α inhibitions therapy in JIA (2). In this study, TNF-a could induce NET formation *in vitro* and the plasma concentration of cf-DNA (NETosis-derived product) was linked to the serum level of TNF-a *in vivo*, implying that TNF-α might augment inflammation by inducing NETosis in JIA patients, then lead to disease progression. ESR is a commonly objective biomarker for monitoring disease activity, and the association of cf-DNA levels with the ESR level further evidenced that cf-DNA concentration was closely associated with disease activity and could be used to monitor disease activity.

Previous work and our data confirmed that TNF-α could trigger NET formation (34) and NET-derived products in turn could act on other immune cells to secrete TNF-α (42). Therefore, there may be a vicious cycle between NETosis and TNF-α. Consequently, inhibiting NETosis in JIA patients could be useful for preventing the deleterious effects of NETs on inflammation. Anti-TNF-α biologics are effective in most JIA patients (2). However, the effect of TNF-α inhibitor therapies on NET release in JIA patients still requires further study. Recently, some research show that infliximab, a TNF-α inhibitor can inhibit NET formation and reduce disease activity in RA patients (43) and in radiographic axial spondyloarthritis patients (44). In this study, *in vitro* inhibition study showed that TNF-α-induced NET formation was substantially suppressed by adalimumab (**Figures 4A, B**). Furthermore, the ability of TNF-α inhibitors, including adalimumab and etanercept, to suppress NET formation significantly improved disease activity in JIA patients (**Figure 4G**).

Given that enhanced NET formation in the three JIA subtypes, the molecular mechanisms of NETosis in JIA patients and TNF-α-induced NETosis should be studied for NET-targeted therapy for JIA. Our study demonstrated that TNF-α-induced NET formation was markedly reduced in the presence of DPI (**Figure 4D**), and ROS production was increased in neutrophils following TNF-α administration (**Figure 5A**), suggesting that ROS generation by activated NADPH oxidases is required for TNF-α-induced NET formation. Several signaling pathways have been found to regulate the activation of NADPH oxidase, and PI3K-dependent signaling is the central pathway, and the blockade of which severely limits the activation of NADPH oxidase (37, 45), we thus examined the effect of TNF-α on PI3K-AKT-NADPH signaling activation and found TNF-α caused a dramatic increase in the phosphorylation levels of PI3K and AKT in neutrophils (**Figure 6A**), indicating that TNF-α-induced NETs might occur through a ROS- and PI3K-Akt signaling-dependent pathways. Notably, NET formation in JIA patients might occur through activation of the PI3K-Akt-dependent and MAPK-ERK signaling pathways, followed by the production of ROS by NADPH oxidase and the subsequent activation of MPO, and CDKs, which was proven by increases in basal ROS production, the levels of phosphorylated PI3K and AKT, phosphorylated MAPK-CDKs and ERK1/2, and MPO expression in JIA-derived neutrophils (**Figures 5B**, **6C, D**). Previous research has elucidated that NET formation can be initiated by various inflammatory cytokines (34) and various stimuli activate different NET-associated signaling pathways (45, 46). NETosis in JIA might occur through the activation of several signaling pathways, which might be closely associated with various stimuli. These findings may explain why NETosis is enhanced in JIA, and aid in the development of drugs that target NETs to improve the treatment of JIA. The molecular mechanism of NET formation in JIA is a preliminary study, which needs further systematic study.

In conclusion, NET formation was augmented in the three subtypes of JIA, i.e., o-JIA, p-JIA and ERA and might exert detrimental roles in JIA. Furthermore, TNF-α may be involved in the immunopathogenesis of JIA by enhancing NET formation. Importantly, NET-derived products: cf-DNA and MPO-DNA complex could be used as potential biomarkers for diagnosis and disease activity.

## Data Availability

The original contributions presented in the study are included in the article/supplementary material. Further inquiries can be directed to the corresponding author.
